# Achievements and challenges in structural bioinformatics and computational biophysics

**DOI:** 10.1093/bioinformatics/btu769

**Published:** 2014-12-08

**Authors:** Ilan Samish, Philip E. Bourne, Rafael J. Najmanovich

**Affiliations:** ^1^Department of Plant Sciences, Weizmann Institute of Science, Rehovot, 76100, Israel, ^2^Ort Braude College, Karmiel, 2161002, Israel, ^3^Office of the Director, National Institutes of Health, Bethesda, MD 20814, USA and ^4^Department of Biochemistry, University of Sherbrooke, Sherbrooke, J1H 5N4, Canada

## Abstract

**Motivation:** The field of structural bioinformatics and computational biophysics has undergone a revolution in the last 10 years. Developments that are captured annually through the 3DSIG meeting, upon which this article reflects.

**Results:** An increase in the accessible data, computational resources and methodology has resulted in an increase in the size and resolution of studied systems and the complexity of the questions amenable to research. Concomitantly, the parameterization and efficiency of the methods have markedly improved along with their cross-validation with other computational and experimental results.

**Conclusion:** The field exhibits an ever-increasing integration with biochemistry, biophysics and other disciplines. In this article, we discuss recent achievements along with current challenges within the field.

**Contact:** Rafael.Najmanovich@USherbrooke.ca

## 1 INTRODUCTION

Structural bioinformatics, originally known as structural computational biology, predates other forms of bioinformatics. It can be argued that the seminal 1953 article by Watson and Crick (Watson and Crick, 1953) is in fact a modeling paper and arguably the first structural bioinformatics paper. Thus, the 2014 Nobel prize for ‘multiscale modeling’ to Martin Karplus, Arie Warshel and Michael Levitt marks an important hallmark acknowledging the impact of structural bioinformatics on science. In his account of the birth of the field, Levitt (2001) describes how computation was required to accurately refine the tRNA model predicted by Crick in building an actual model that was taller than himself. Thus, computation has been an integral part of structural biology from its early days and has had an ever-increasing role in biochemistry and molecular biology with the passing of years. Indeed, from the first simulations of small systems and a few picoseconds acknowledged by the Nobel committee, we are now at a stage where millisecond simulations (Beauchamp *et al.*, 2011) or massive searches of sequence and structure space as required for, e.g. computational protein design (Gu and Bourne, 2011; Kiss *et al.*, 2013; Samish *et al.*, 2011) are achievable.

Structural bioinformatics or structural computational biology, broadly defined, is a field at the intersection between computer science, physics, chemistry and molecular biology. Historically, the term ‘structural bioinformatics’ describes data-driven statistical, knowledge-based research of representative non-redundant ensembles of structures to understand the statistical behavior of the system under investigation. Alternatively, ‘computational biophysics’ describes a hypothesis-driven physics-based treatment of biological molecular systems. The ergodic hypothesis guarantees that conclusions from the two types of approaches converge over large non-redundant samples or long simulations (Samish, 2009). Currently, numerous methodologies employ ideas from both approaches. Consequently, hereafter we will refer to both as structural bioinformatics.

Biologically, structural bioinformatics aims to understand the factors that influence and determine the function of biological macromolecules, the interplay between evolution, kinetics and thermodynamics, the determinants of specificity and selectivity in molecular interactions, the dynamic aspects of macromolecular structures and their effect on function and stability and, finally, the ability to use all these for engineering, design and biotechnology. In fact, a complete understanding of biological processes must inescapably pass through an understanding of the factors influencing such processes at the atomic and sometimes even subatomic levels. In this article, we discuss some of the most notable achievements in structural bioinformatics over the past 10 years and discuss existing challenges in the field. Without a doubt, the topics and the specific articles mentioned here are biased by the opinion of the authors.

## 2 ACHIEVEMENTS

**Fig. 1.**
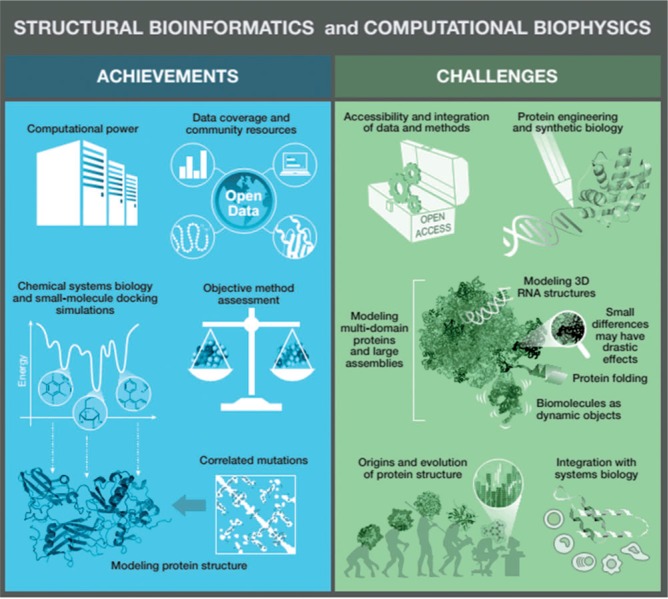
Schematic representation of the main achievements and challenges in the field of structural bioinformatics and computational biophysics as discussed in the text

Some of the numerous achievements in the field in the last 10 years include the following (Fig. 1).

### 2.1 Data coverage and community resources

The human genome sequencing revolution marks the availability of sequence data at scale. The realization that sequence alone is not enough to understand/predict function led to the establishment of large structural genomics initiatives where the structure of proteins with low sequence similarity to proteins with known structures were targeted to increase the coverage of fold space and permit more accurate fold recognition, threading and homology modeling (Baker and Sali, 2001). In the last decade, the number of structures deposited in the protein data bank (PDB) (Berman *et al.*, 2000) grew 4-fold to over 100,000 structures including an unprecedented number of membrane protein structures. The PDB—one of the first biological databases—and derived resources such as CATH (Sillitoe *et al.*, 2013), SCOP (Andreeva *et al.*, 2008) and PFAM (Finn *et al.*, 2014) are fundamental enabling tools for the entire field and their long-term maintenance is of great importance to the field.

### 2.2 Computational power

The increased availability of computer power has enabled applications that were beyond imagining just a few years ago. Dedicated hardware [e.g. ANTON (Shaw *et al.*, 2009) at DE Shaw Research designed uniquely for molecular dynamics simulations], the use of graphical processing units (Friedrichs *et al.*, 2009) or distributed crowd sourcing with the BOINC interface (Anderson, 2004) [e.g. Folding@home (Shirts and Pande, 2000)] vastly increased the reach of current methods. An exciting development is the involvement of interested laypeople as active problem solvers such as in the Foldit game (Cooper *et al.*, 2010).

### 2.3 Objective method assessment

The CASP critical assessment of protein structure prediction began a new era in the field providing an objective double blind test for structure prediction methods (Moult *et al.*, 2014) with other areas following suit: protein interactions (Janin *et al.*, 2003), function prediction (Radivojac *et al.*, 2013), membrane protein docking (Kufareva *et al.*, 2014) or automated structure prediction (Bourne, 2003). This also led to meta-prediction methods (Ginalski *et al.*, 2003) and community collaborations, e.g. WeFold (Khoury *et al.*, 2014).

### 2.4 Correlated mutations and modeling protein structure

Correlated mutation data have enabled the generation of pairwise amino acid contact maps from sequence data (Marks *et al.*, 2011; Nugent and Jones, 2012). Such contact maps are then used as spatial constraints to generate models of globular and membrane proteins. In the cases where enough sequence data allow the use of this methodology, a high level of accuracy has been achieved.

### 2.5 Chemical systems biology

Also known as systems pharmacology, the integration of the vast amounts of ‘omics’ data with accessible structural methods such as the detection of binding site similarities (Kurbatova *et al.*, 2013; Xie *et al.*, 2011a) can be used for drug repositioning and discovery (Chang *et al.*, 2010; Kinnings *et al.*, 2009; Xie *et al.*, 2011b, 2014).

### 2.6 Small-molecule docking simulations

Docking simulations (Brooijmans and Kuntz, 2003; Halperin *et al.*, 2002) are widely used (Kitchen *et al.*, 2004; Warren *et al.*, 2006). Predicted ligand protein complex structures are then used to generate hypotheses about binding and for virtual screening in the early stages of drug design.

## 3 CHALLENGES

Although considerable progress has been made, advances are still needed in the following areas (Fig. 1).

### 3.1 Modeling large or multi-domain proteins and assemblies

Most proteins are large and multi-domain and take part in complex assemblies requiring fine-tuned recognition (Chothia and Janin, 1975; Jones and Thornton, 1996; Levy *et al.*, 2008). Examples include large complexes such as the ribosome (Yonath, 2010) and the proteasome (Adams, 2003) or supercomplexes such as the 60-subunit pyruvate dehydrogenase complex (Patel *et al.*, 2014). Most of cell biology requires understanding the interplay of such large multi-domain proteins and complexes and lacks the level of detail that comes from atomic-level structural information. Targeting such complexes and assemblies experimentally and looking at them in the context of the complete cell is an emergent challenge.

### 3.2 Biomolecules as dynamic objects

The accurate modeling of large conformational changes due to ligand binding, allosteric effects, post-translational modifications or as the result of protein–protein interactions is essential. Techniques such as molecular dynamics (Adcock and McCammon, 2006) and elastic network models (Bahar and Rader, 2005; Frappier and Najmanovich, 2014) provide two techniques at different granularities. Descriptions of biomolecules representing the conformational ensembles in which they are found under physiological conditions will expand our understanding of the relationship between structure and function. The use of such conformational ensembles as opposed to single static structures is challenging for complex structures.

### 3.3 Modeling 3D RNA structures

The prediction of RNA structures is still in its infancy (Laing and Schlick, 2010; Rother *et al.*, 2011) and often requires processing low-resolution data (Parisien and Major, 2012). With simplified rules for molecular interactions in RNA when compared with proteins, we should be able to model larger and more complex RNA molecules than currently possible.

### 3.4 Small differences may have drastic effects

Although protein structure is resilient to mutation (Baase *et al.*, 2010), function is not necessarily as resilient (Najmanovich *et al.*, 2008; Rajamani *et al.*, 2004). This combination of structural robustness and functional plasticity is at the core of evolutionary change. It is essential that we learn to recognize and accordingly weigh such functional determinants to predict the outcome of natural or engineered perturbations at the molecular level. In particular, the prediction of function is based on the detection of similarities (Najmanovich *et al.*, 2005) where small differences are ignored. One needs to only look at the existence of vast protein families performing the ‘same’ function to realize that we do not fully understand the effect of small differences. Some of these differences are likely required to modulate selectivity rather than specificity within the different cellular (or temporal) contexts where the same function is required (Najmanovich *et al.*, 2008). This level of understanding will require the integration between structural and systems biology.

### 3.5 Integration with systems biology

Beyond the detection of cross-reactivity targets to a given drug and the impact on the complete system, a full understanding of specificity and selectivity at the molecular level requires studying all macromolecules that are sharing the crowded (Ellis, 2001; McGuffee and Elcock, 2010; Minton, 1993) cellular milieu. Computationally understanding a full cell from a structural point of view is a major challenge. A full structural understanding of a living cell at all scales (Stein *et al.*, 2007), integrating macromolecules at atomic resolution all the way to phenotypes is still missing and once achieved will define a new era in biology (Hallock *et al.*, 2014; Peterson *et al.*, 2014; Winter *et al.*, 2012).

### 3.6 Protein engineering and synthetic biology

Protein engineering of single proteins (Kiss *et al.*, 2013) and protein–protein interfaces (Potapov *et al.*, 2008) is an advancing field, in particular with the success of Rosetta (Rohl *et al.*, 2004) and the advent of a whole generation of ‘Rosetta Engineers’ (The term was heard at the Protein Engineering Canada 2014 conference but the author is unknown.). However, computational protein design (Samish *et al.*, 2011) and enzyme redesign (Gerlt and Babbitt, 2009) are still restricted by the complexity of the sequence and structure search space. The approximations that are needed to tackle this complexity often require a choice of simplified methods that all but ignore atomic structure. Specifically, side-chain placement, iterative homology modeling (Q. Wang *et al.*, 2008) and flexible backbone sampling (Ollikainen *et al.*, 2013) remain major challenges. Lastly, synthetic biology (Way *et al.*, 2014) promises to extend biological systems well beyond what is naturally observed and its integration with computational drug design (Marchisio and Stelling, 2009; Tew *et al.*, 2010) and structural bioinformatics will open new and exciting possibilities.

### 3.7 Origins and evolution of protein structure

Is fold space discrete or continuous? If discreet, why are some folds so much more common than others? Have we identified nearly all possible folds? How do new folds appear? All these questions have been partially addressed but definitive answers still remain illusive.

### 3.8 Protein folding

The biggest challenge in structural bioinformatics remains unsolved. That is, the ability to consistently predict the structure of a protein based solely on its amino acid sequence. The Levinthal paradox inspires us to continue. Thus, although conformational space is so immense as to be intractable, proteins do fold, leading to a variety of advances based on different ways of addressing the paradox (Dill and Chan, 1997; Karplus, 1997; Wolynes *et al.*, 1995). However, we are far from solving the protein folding problem and arguably progress is based more on existing structures than first principles.

### 3.9 Accessibility and integration of data and methods

Data, methods and publications must be open (Masum *et al.*, 2013) and reproducible (Sandve *et al.*, 2013). One success of the field has been the number of mature open source software. Widely used software includes CCP4 for macromolecular structure determination and refinement (Potterton *et al.*, 2004), CHARMM (Hynninen and Crowley, 2014), NAMD (Y. Wang *et al.*, 2011) and GROMACS (Pronk *et al.*, 2013) for molecular dynamics, Modeller (Eswar *et al.*, 2007) for homology modeling and Rosetta (Rohl *et al.*, 2004) for structure prediction and design. One challenge for the future is how to make the plethora of existing methods accessible to newcomers in the field and to the scientific community at large. Just as one cannot publish a structure or a sequence without submitting the data to a public repository, methods and data must be stored in repositories that guarantee their accessibility to the community immediately at the time of publication. The availability of data and methods will help ensure reproducibility and cross-validation.

## 4 3DSIG

3DSIG is a special interest group within the International Society for Computational Biology. 3DSIG holds an annual meeting preceding the annual ISMB meeting. The insights shared in this article come as a synthesis of the trends observed at 3DSIG since its inception 10 years ago.
